# Baron Alderson's "Charge" against Private Lunatic Asylums

**Published:** 1852-07-01

**Authors:** 


### BARON ALDERSON'S "CHARGE" AGAINST PRIVATE LUNATIC ASYLUMS.

Accustomed as we are to receive with habitual respect and reverence the opinions enunciated by the learned judges of our land in summing up evidence upon an infinite variety of judicial points, we confess it gives us pain whenever we feel ourselves called upon to demur against their authority in matters connected with this department of our profession. Only recently the *dictum* of Lord Chief Baron Pollock, in the celebrated case of "Nottidge *v.* Ripley," that all persons apparently "harmlessly insane," no matter under what delusions they might labour, were improper subjects for confinement in lunatic asylums, and ought immediately to be discharged, called forth the remonstrances of the commissioners in lunacy; and the utopian doctrine of the learned judge has, we believe, fallen to the ground. Not less erroneous is an opinion just promulgated from the bench by Mr. Baron Alderson, which challenges the most peremptory refutation. On the 16th instant, one Henry Baker surrendered at the Central Criminal Court, to take his trial upon a charge of misdemeanour, for having kept two or more lunatic persons in his house, such house not being licensed according to the act of parliament. In consequence of information received by the commissioners in lunacy, Mr. Law, their solicitor, accompanied by the Earl of Shaftesbury and other gentlemen, went to the house of the defendant, in Doris Street, Lambeth, and they found him guilty of the charge preferred against him.

There was, therefore, no alternative, excepting for his counsel, Mr. Ballantine, to admit that he had acted illegally, and to sue for mitigation of sentence. On the part of the prosecution, Mr. Clarkson stated that the object of the commissioners was to prevent a proceeding which was undoubtedly illegal and improper. This was the fourth case of the same kind which they had felt it their duty to bring forward; and all they required in this case was, that the offending parties should enter into sureties not to repeat the offence. There cannot, as we shall have occasion to explain, be the least doubt of the propriety of this prosecution; and the spirit of forbearance with which it was conducted is manifest; when, however, the defendant had pleaded "guilty," it appears to us that all that remained for the court to do was to convict, and fix the amount of recognizances required. But Baron Alderson did not stop here; he travelled out of his judicial capacity, and denounced in general terms the existence of all private lunatic asylums. We quote *verbatim* his observations, as reported in the "Times" of June 17. "Mr. Baron Alderson said, that many very *good and sensible persons* had entertained the opinion that *there ought to be no private lunatic asylums—but that they should all be public.* He must confess that this was his own opinion, as he could not help thinking that it was a very dangerous practice to allow persons to have the charge of lunatics *who might have a personal interest in keeping them in that miserable condition. It would be very easy to have private wards in a public asylum, and in such an establishment no inducement of the description he had alluded to could possibly exist. He himself should certainly not like to be placed in one of these private establishments; and, therefore, he felt their impropriety for the reception of others.*" The case was then disposed of, by the defendant being directed to enter into a personal recognizance of 1000*l.*, and find two sureties in 100*l.* each, to appear and receive judgment, should he be called upon to do so.

Now, we would fain ask what was the question involved in this trial? Assuredly, not the existence or non-existence of private lunatic asylums. The very prosecution itself was founded in vindication of the law which governs these establishments. Here we have a man who was previously one of the subordinate officers in Bethlem Hospital, availing himself, probably, of the knowledge he there acquired in his capacity of a servant; he then leaves that institution, and goes and opens a house of his own; in the phraseology of the vulgar, "sets up in business for himself." Accordingly he takes, be it observed, a small house in an obscure suburb of the town, never having applied to the commissioners for a licence, and, thus evading the law, he carries on his trade in secret safety; the commissioners having no knowledge of the existence of such a place, he could treat his patients in any way he pleased, they being subjected to no manner of surveillance. This was, therefore, a very proper case for indictment, the charge being materially aggravated by the fact of the man having been an officer in Bethlem. The proprietors of Private Lunatic Asylums too frequently find to their cost, their officers, attendants, and servants attempting to play these pranks; and when they are detected and convicted, although it be well that mercy should temper justice, they ought not to escape quite so lightly as did this Henry Baker! But we are at a loss to discover what feature or circumstances of the case could suggest, or justify, the above unprovoked philippic against Private Lunatic Asylums? Assuredly, the learned baron confounded *un*licensed with licensed houses; he had in his mind's eye the house of Henry Baker, not asylums legally licensed and under the immediate jurisdiction of the commissioners. "Many very good and sensible persons" as he calls them, may entertain very erroneous opinions on many subjects, and if they were acquainted with the practical working of Public Lunatic Asylums, they would know well the inexpediency of wards being established in such establishments for the reception of private patients. Many years ago Lord Monteagle, in his evidence before the House of Lords, described the many inconveniences and evils which would arise from private patients being admitted into pauper asylums; and so convinced of this fact are the visiting justices, who know well the system which is necessary for the management of county asylums, that many of them, particularly in the North of England, have come to the resolution not to grant any establishment in future a licence for the two classes. They know, practically, that the better class of persons afflicted with insanity, are best taken care of in private asylums, where the various domestic appointments and arrangements correspond with those to which they have been accustomed; they know, also, that educated persons require attention, and a variety of comforts in accordance with their previous habits, which pauper patients never dreamt of. It may be very well for the learned Baron Alderson, in a sound and vigorous state of mind, to think he would prefer for himself a "private ward in a public asylum,"—were he, Heaven forbid! ever to be so afflicted. But we can tell his lordship that educated patients, those who have moved in the middle as well as in the upper classes of society, when they become mentally afflicted, evince an intense dread of being domiciled, notwithstanding any arrangements that may be devised for their complete separation, with paupers. To a refined and sensitive mind the very idea has a most depressing influence. They do not meet them, it is true, in the wards or corridors; but they see them from the windows; they meet them, perhaps, in the open ground, and they know and feel they are breathing the same atmosphere. The public generally,—the relations and friends of patients,—also strongly object to the plan—a fact well known to the medical men at Manchester, who wish to support Cheadle, an establishment intended for the reception of private as well as pauper lunatics. Apart, however, from all moral considerations, the management adopted in public and in private asylums must ever be essentially different. Hanwell Asylum with above 900; Lancaster with nearly 800; and Surrey with above 700 pauper patients; indeed, any large county asylum, would be seriously inconvenienced by the introduction of private wards. The practical difficulties, where the plan has been tried, have been found to be insuperable!

To conclude, we once more emphatically repudiate the insinuation, that persons who have the charge of lunatics," from having "a personal interest in keeping them," can be base enough to prolong their "miserable condition." The prosperity of an asylum depends entirely on the public confidence it enjoys; and the interest of the proprietor, if we condescend to meet the accusation upon the most selfish ground, is obviously to prove to the relatives or friends of the patient, that everything possible is being done to ameliorate his or her condition, and accelerate recovery. Upon no other principle can we understand any asylum being conducted. The self-same charge, may, however, be brought against the managers of public asylums; they have an exchequer to maintain; they have an interest in keeping up the full complement of patients in the house; they must keep their revenue up at *par;* and to satisfy the grumbling rate-payers, they must have their contracts cut down to the most transparent shaving; they also must exhibit a satisfactory amount of receipts upon their balance-sheet. They, therefore, have a "personal interest," as the learned baron calls it, in taking charge and keeping pauper lunatics, quite as strong as might be supposed to actuate any unworthy proprietor of a private lunatic asylum. Imputations of this description, whether they emanate from the bench or from the bar, are very unjustifiable. There is no profession or occupation in life in which pecuniary compensation does not afford a very proper and healthful stimulus to honourable exertion. The ministers of the crown—the bishops of the church—the judges of the realm—the learned baron himself—never, we feel assured, disdained their drafts upon the treasury, or the bank of England; but to impeach their motives, because they enjoy the possession of a large revenue, would be as unjust as it is to impute such conduct as Baron Alderson has done to those who are engaged in the arduous and anxious duties of a profession not less honourable, although perhaps not so distinguished, as any of the above.

---

